# Book Review: Los invertebrados de hábitats subterráneos de Jaén: By Pérez-Fernández, T. and Pérez-Ruiz, A

**DOI:** 10.3897/zookeys.518.9684

**Published:** 2015-08-25

**Authors:** Francisco Javier Peris-Felipo

**Affiliations:** 1 Bleichestrasse 15, CH–4058, Basel (Switzerland)

This book concerns the review of both the history of speleology in southern Spain and the invertebrate fauna of Jaen province within two parts: (1) ‘Historia de la Espeleología en Andalucía’; and (2) ‘Medio subterráneo y organismos hipogeos’. First contributions were carried out very early in the 20th century by Abbé Henri Breuil, Pierre-Jules Rambur, Lucas von Heyden, and Georg Dieck. However, until the late 1940s and early 1950s, no more studies were attempted. From this time, different researchers such as Joaquín Mateu, Antonio Cobos, and Dr Francesc Español, begun to study the area and increase research output. During the 1970s to 1990s, many entomologists and biospeleologists belonging to different societies sampled in the Andalusian caves. At the beginning of the 21st century, amateur societies such as ‘Grupo de Espeleologia de Villacarrillo’ (G.E.V.), ‘Espeleo Club Almería’, ‘Grupo de Exploraciones Subterráneas de la Sociedad Excursionista de Málaga’, ‘Grupo de Espeleólogos Granadinos’, ‘Grupo de Exploraciones Subterráneas de Priego’, ‘Grupo de Investigaciones Espeleológicas de Jerez’ (G.I.E.X.), and ‘Club Deportivo Plutón’ continued exploring the caves and increasing our knowledge of hypogeal species from southern Spain.

**Figure:**
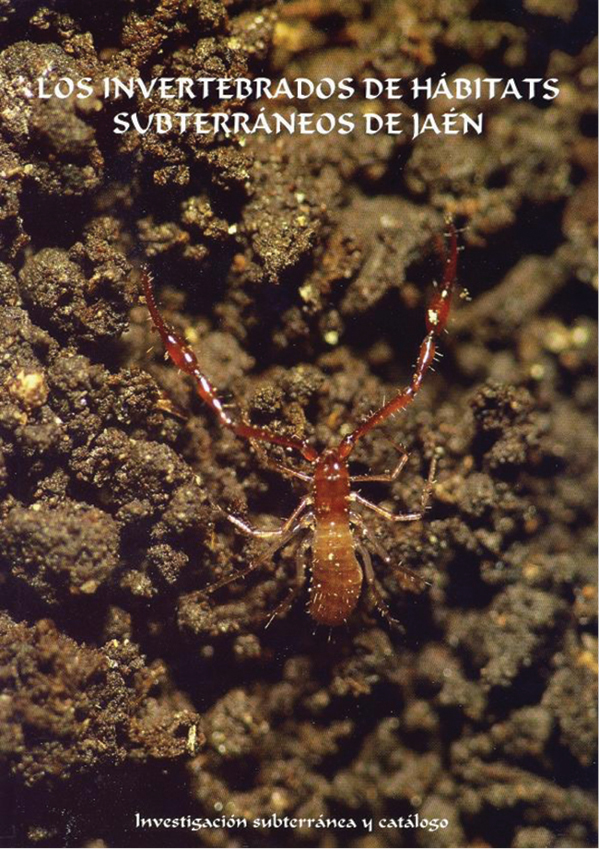
“Los invertebrados de hábitats subterráneos de Jaén”

In the second part, an explanation of the ecological subterranean landscape and the list of recorded hypogeal species from Jaen province are provided in 26 chapters. The authors list them by phylum, order, family, and species. Additionally, short descriptions of each species, its distribution, habitat, and/or some pictures are provided.

A total of 290 hypogeal species are listed including the phyla Annelida (2), Chelicerata (41), Crustacea (18), Hexapoda (148), Mollusca (16), Myriapoda (23), and Nematoda (42). These species belong to 31 orders; among them, Coleoptera is the most abundant. Also, 31 species were recorded for the first time in Andalucía: *Ablechroiulus spelaeus*, *Acipes andalusius*, *Atheta tenebrarum*, *Camaeus gevi*, Chthonius (Ephippiochthonius) cazorlensis, Chthonius (Ephippiochthonius) espanyoli, Chthonius (Ephippiochthonius) giennensis, Chthonius (Ephippiochthonius) perezi, Chthonius (Ephippiochthonius) villacarrillo, *Ceratosphys jabaliensis*, *Coletinia tinauti*, *Corynoptera latibula*, *Domene cavicola*, *Domene perezi*, *Habrocerus ibericus*, Laemostenus (Antisphodrus) cazorlensis cazorlensis, Laemostenus (Antisphodrus) cazorlensis divergens, *Nemastomella gevia*, Neobisium (Ommatoblothrus) espinoi, Neobisium (Ommatoblothrus) perezi, Neobisium (Ommatoblothrus) perezruizi, *Nesticus baeticus*, Petaloptila (Zapetaloptila) carabajali, Petaloptila (Zapetaloptila) mogon, *Protonemura gevi*, *Pseudosinella baeticaense*, *Psichrosoma baeticaense*, *Pygmarrhopalites perezi*, *Stegelletina coprohila*, *Tinautius troglophilus*, and *Trichoniscus perezi*.

To conclude the book, the authors provide information about the distribution of caves with a complete listing and map. This book is essential reading to increase and understand to the history of Andalusian speleology and, especially, to acquire knowledge of species that live in these somewhat surprising habitats.

2013, Grupo Espeleología Villacarrillo, Jaén, 188 p., Deposito Legal: J 448-2013

23.6 × 16.9 cm, colour, 13 € ($ 14).

